# Supplementary material for: The Effect of the Lysine Acetylation Modification of ClpP on the Virulence of Vibrio alginolyticus
Source: Molecules. 2024 Sep 9;29(17):4278. doi: 10.3390/molecules29174278 (PMC11396845; doi:10.3390/molecules29174278)
Supplement: Supplementary file 1 [file molecules-29-04278-s001.zip › molecules-3123532-supplementary.pdf]

**Table S1** Sequences of primers used in this study.

| Primers | Nucleotide sequence (5′-3′) | references |
|---|---|---|
| *clpP*-F | ATGAGCTACCAAGAAAAAAATGCAA | This study |
| *clpP*-R | TTACTCGCCACGGTGGCTTAACACT | This study |
| pET-28a-*clpP*-F | CGCGGATCCATGAGCTACCAAGAAAAA | This study |
| pET-28a-*clpP*-R | CCGCTCGAGCTCGCCACGGTGGCTTAA | This study |
| pET-28a-YZ-F | TGCTAGTTATTGCTCAGCGG | This study |
| pET-28a-YZ-R | TAATACGACTCACTATAGGG | This study |
| *clpP*-com-F | agtggatcccccgggctgcagACCAGAACTAAACGACGAGTTCG | This study |
| *clpP*-com-R | ggtaccgggccccccctcgagCCAAAATGCGCTGGCTAATT | This study |
| pBBR1-YZ-F | ggatcttccagagatATGTGCTGCAAGGCGATTAAG | This study |
| pBBR1-YZ-R | ctgccgttcgacgatTGACCATGATTACGCCAAGCG | This study |
| K165Q- *clpP*-F | CcagTTGTTAGCAGAACATACAGGCCAACCTC | This study |
| K165Q- *clpP*-R | GTTCTGCTAACAActgGTTTAGCTTCTGTTTAATCGTCAGGA | This study |
| K165R- *clpP*-F | CaggTTGTTAGCAGAACATACAGGCCAACCTC | This study |
| K165R- *clpP*-R | GTTCTGCTAACAAcctGTTTAGCTTCTGTTTAATCGTCAGGA | This study |
| K165A- *clpP*-F | GCTAAACgcgTTGTTAGCAGAACATACAGGCCA | This study |
| K165A- *clpP*-R | CTAACAAcgcGTTTAGCTTCTGTTTAATCGTCAGGA | This study |
